# The Effect of Oral Protein Supplementation on the Growth of Very Low Birth Weight Preterm Infants Admitted to the Neonatal Intensive Care Unit: A Randomized Clinical Trial

**DOI:** 10.34763/jmotherandchild.20232701.d-22-00072

**Published:** 2023-06-27

**Authors:** Fariba Hemmati, Maral Ghassemzadeh

**Affiliations:** Associate Professor of Pediatrics and Neonatologist, Neonatal Research Center, Department of pediatrics, School of Medicine, Shiraz University of Medical Science, Shiraz, Iran; Assistant Professor of Neonatology, Tehran University of Medical Sciences, Hakim Children Hospital, Tehran, Iran

**Keywords:** Protein supplement, Preterm infants, Growth, Nutrition, VLBW

## Abstract

**Background:**

During NICU admission, extra-uterine growth retardation that can affect the neurodevelopmental outcome is a challenging problem in extremely preterm infants. This trial aimed to determine the effect of additional enteral protein supplementation on the growth velocity of the anthropometric parameters.

**Method:**

In this randomized controlled trial, 77 preterm infants (gestational age ≤33 weeks and birth weight <1500 g) who reached full enteral feeding with either fortified breast milk or preterm formula were included. They were randomized to receive either 4-<5 g/kg/day protein through extra protein supplementation (intervention) or 3-<4 g/kg/day protein. Weight gain, as well as length and head circumference growth, were monitored daily and weekly, respectively. Venous blood gas, blood urea nitrogen (BUN), and albumin levels were checked weekly.

**Results:**

Five out of 77 participants were excluded due to feeding intolerance. Analyses were conducted on 36 neonates with protein intake of 3.66 ± 0.22 gr/kg/day and 36 with extra protein intake. Baseline characteristics were similar between the groups. An additional protein supply of 0.89 gr/kg/day, resulting in an average protein intake of 4.55 ± 0.18 in the intervention group, increased the postnatal weight gain, linear growth, and head circumference growth (7.98 gr/kg/day, 0.347 cm/week, and 0.38 cm/week, respectively). The albumin levels were significantly increased, but the BUN levels were not significantly increased in the intervention group. None of the patients developed necrotizing enterocolitis or significant acidosis.

**Conclusion:**

Protein supplementation significantly improves the growth of the anthropometric parameters. An increase in serum albumin and no increase in serum urea can indicate the anabolic effect of extra protein. Protein supplementation can add to routine feeding protocols of VLBW infants without any short-term adverse effect; however, further study for evaluation of long-term complications is needed.

## Introduction

The last trimester of pregnancy, which is often lost in preterm birth, is the time when the fetus has the most protein intake for growth. Furthermore, extremely preterm infants lose protein approximately twofold that of term infants [1]. Therefore, preterm newborns often experience a negative nitrogen balance. Prematurity is a nutritional emergency since studies demonstrate that these infants experience extra-uterine growth retardation as a result of a cumulative deficiency in protein and calorie intake [2]. Postnatal growth failure (PNGF) has been defined as weight less than the 10th percentile for gestational age (GA). The incidence of PNGF reported by the neonatal research network of National Institute of Child Health and Human Development (NICHD) [3] and in Ethiopia [4] was as high as 79% and 86%, respectively. Ehrenkranz et al. reported the association between weight gain in the neonatal intensive care unit (NICU) and neurodevelopmental outcomes in a cohort of 600 extremely low birth weight infants [5]. They divided the infants into four groups based on growth velocity during NICU admission; infants in the highest quartile gained an average of 21 g/kg per day, and those in the lowest quartile gained 12 g/kg per day. Neurodevelopmental impairment was significantly higher in infants in the lowest quartile of weight gain than those in the highest quartile. Similar findings were also observed with head growth during admission. Likewise, Ramel and colleagues have described the association between poor linear growth and neurodevelopmental outcomes [6]. As evidence continues to accumulate that PNGF has long-term consequences, optimizing the provision of nutrition in VLBW neonates is critical to guarantee the best potential outcomes.

Over the past decade, the incidence of preterm births and survival rate of very low birth weight (VLBW) infants has increased. The medical care and survival rates of preterm infants, even those as early as 24 weeks GA and as small as 600 g, have improved remarkably [7]. Optimal nutrition of these preterm infants should be unique, and it must provide growth that mimics normal fetal growth. Although the growth of preterm infants is usually impaired by frequent pathophysiologic events, pharmacological treatment, and physiologic immaturity, protein intake is critical for preterm infants’ growth. According to the recommendation of the American Academy of Pediatrics (AAP), enteral protein intake in preterm infants should be 3.5–4 gr/kg/day [8]. However, the recommendation of European Society for Pediatric Gastroenterology Hepatology and Nutrition (ESPGHAN) for protein intake in infants weighing up to 1000g is 4.0–4.5 g/kg per day and 3.5–4.0 g/kg per day for infants weighing 1000–1800gr [9]. An international consensus panel has also recommended 3.5–4.5 g of protein per kg/day for VLBW infants receiving full enteral feeding [10]. ESPGHAN and AAP both advise the mother's own milk (MOM) as the primary choice for feeding preterm newborns. If MOM is not accessible, the use of pasteurized donor human milk has been recommended [11]. If both of them are unavailable, preterm formula (PF) should be used. Preterm formulas, developed to meet the nutritional needs of growing preterm infants, contain more protein than term formulas. The protein content of standard PF and “high protein” PF are 3 g/100 kcal and 3.3–3.6 g/100 kcal, respectively [12].

Although the protein content of preterm human milk is greater than term human milk, it does not entirely provide the nutritional needs of preterm infants. The protein content and composition of human milk change through the first weeks of lactation from approximately 1.7 g/dL at 7 days to 1.2 g/dL by 42 days [12]. Human milk fortifiers have been developed to add the protein content and insufficiency of human milk; however, expectations about the final protein content of fortified preterm human milk must be challenged due to steady decline in the protein content as lactation progresses. Corvaglia and colleagues showed that standard fortification failed to meet the recommended protein intake by bedside near-infrared-reflectance-analysis of milk [13]. Standard fortification methods presume that breast milk is uniform in protein content. In adjustable fortification, however, additional protein supplementations in the intervention group were changed weekly according to blood urea nitrogen (BUN) levels to reach the goal level [14]. Targeted fortification, which is another method of individualized fortification, is dependent on the analysis of breast milk macronutrient content and the amounts of protein supplement is changed based on MOM [15]. Ergenekon et al. assessed the short and long-term effects of individualized enteral protein supplementation in 33 preterm newborns with GA≤32 weeks and reported that standard preterm nutrition with fortified Breast milk (FBM) may not be sufficient, and additional enteral protein supplementation improved the physical growth rate in the NICU and improved neurodevelopmental outcome at 18 months’ corrected age [16]. Conversely, Maas and colleagues reported that increase in protein intake to a mean intake of 4.3 g/kg/d did not further enhance the growth of very preterm infants with a median birth weight of 1200 gram [17]. In summary, there are controversies in preterm feeding protocols and further studies are required.

The objectives of this prospective clinical trial are to determine the growth parameters during the NICU admission in preterm infants with GA≤33 weeks and birth weight less than 1500 g (VLBW) and evaluate the effect of additional enteral protein supplement on the anthropometric parameters after reaching full enteral feed with either FBM or PF; we also aimed to check the effect of high protein milk on albumin, BUN levels and venous blood gas analysis, and incidence of NEC.

## Patients and methods

This double blind randomized controlled (RCT) trial was conducted at Nemazi and Hafez hospitals affiliated to Shiraz University of Medical Science, Iran, from Feb 2017 to April 2018. All stable preterm infants with birth weight less than 1500 g and GA≤33 weeks who were admitted to these NICUs were assessed for eligibility and enrolled after fulfilling the inclusion criteria. Each preterm neonate who reached full enteral feeding (150–180 ml/kg/day) with either FBM or PF was included. The exclusion criteria of the study were refusal by either parents or neonatologist, presence of severe congenital anomalies especially gastrointestinal anomaly, intraventricular hemorrhage grade III or more, hydrocephaly, periventricular leukomalacia, large PDA with signs of heart failure, sepsis, surgery, systemic steroids use, and persistent oral feeding intolerance during the study. Feeding intolerance was managed by temporary interruption of human milk fortifier or protein supplement or formula, but if intolerance was repeated more than 3 times, the infant was excluded from the study.

The participants were divided into two groups. One group received protein supplements (intervention group), while the other group did not (control group). We used a randomized sampling method via blocked randomization. Both the parents and the data collector were unaware of the group allocation. The only individuals who were aware of the protein supplement allocations were a research fellow and a nurse who were not involved in the measurements.

All participants received 3 g/kg/day parenteral protein as Aminovein infant 10% (FRESENIUS KABI Company, Germany) from the first hours of life and gradually increased to 3.5 g/kg per day. Enteral feeding started as early as possible. The preferable feeding method was breast milk. When the total volume of breast milk reached 120 ml/kg/day, human milk fortifier powder (Aptamil FMS, DANONE company, Netherlands) was added and increased daily to its complete dosage of 4 scopes per day (4.4 g) which contain 1.1 g protein. When the neonates received 150–180 ml/kg/day FBM, they were considered as full fed. By considering the amount of protein of breast milk as an average of 1.5 g/dl [12], the amount of protein intake in infants feed FBM was 3.35–3.8 g/kg/day. When breast milk was not available, preterm formula started and gradually increased to 150 ml/kg/day. By calculating the amount of protein content of different PFs and one special formula (for example 2.3 g/dl in pre-nan, 2.6 g/dl in premature BeBelac and in PRE-Aptamil formula, and 1.6g/dl in BeBelac anti regurgitation), the amount of protein intake in infants who were fed only preterm formula was 3.4–3.9 g/kg/day. The research fellow calculated the precise amount of protein intake for each neonate; for neonates who were in the intervention group, protein supplement with a dosage of 0.5–1g/kg/day protein (each gram of powder has 0.8 grams protein, Aptamil brand, DANONE company, Netherlands) was added, so that total protein intake of each neonate never exceeded 5 g/kg/day. Each gram of protein supplement was added in each 100ml of FBM or PF. Therefore, the intervention group received 4 to less than 5 g/kg/day protein, whereas the control group received 3 to less than 4 g/kg/day protein. Albumin, BUN, and blood gas levels were checked before starting the study and repeated weekly during the trial. The participants were observed for signs and symptoms of necrotizing enterocolitis (NEC) during the study.

All the neonates were evaluated for daily weight gain with a seca345 scale with 5-gram accuracy. They were also evaluated weekly regarding their head circumference (HC) and linear growth velocity by the special nurse, and the results were recorded as cm per week. Data were collected in predesigned forms with codes assigned to each patient to preserve the patients’ confidentiality and blindness of the study. We used the Fenton curve calculator online from the site peditools.com for calculating the exact percentile of anthropometric measurements. The special nurse was not aware of the case or control groups.

### Ethical considerations

Written informed consent was obtained from the parents before the randomization of the newborns in either group. They were provided with written and verbal information including the potential risk and benefits involved in the research. Parents were informed about the voluntary nature of participation in the research, about non-penalization in the event of nonparticipation, and about the option of withdrawing their infant from the study at any point they wished without offering any reason. Confidentiality of the subjects was maintained.

Permission to carry out the study was sought from the respective university administrators, and the study was conducted in compliance with the relevant guidelines and regulations and the Declaration of Helsinki; the study was also approved by the Medical Ethics Committee of the university (IR.SUMS.MED.REC.1396.107) and registered in the registry of clinical trials (www.irct.ir; Code: IRCT20180923041095N1).

### Statistical analysis

The sample size was calculated based on Agostoni et al.'s study [9] with a confidential interval of 95%, power of 80%, d = 2, and equal variance with a size of 3; a sample size of 36 patients in each group seemed to be sufficient. All data were entered in SPSS, version 26. After evaluation of the normal distribution of the data with the Kolmogorov-Smirnov test, quantitative data were expressed as mean and standard deviation (SD), while qualitative data were expressed as frequency and percentage (%). Comparison of the quantitative data between the two groups was done by an independent sample T-test, while qualitative data were analyzed using a Chi-square or Fisher's exact test. The repeated measures analysis of variance test was used to evaluate the changes in laboratory data throughout the study. A p-value of less than 0.05 was considered statistically significant.

## Results

**Figure 1.**
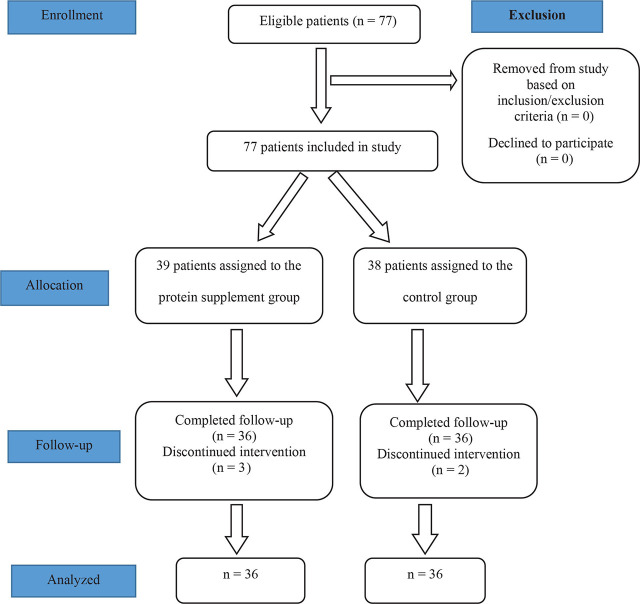
The CONSORT flow chart of the study population.

In this prospective RCT, 77 preterm infants were assessed for eligibility. Five of them were excluded from the study due to feeding intolerance. Three of them were in the intervention group and received protein supplement, and two of them were in the control group. The difference between the two groups according to feeding intolerance was not statistically significant (P=1.000). Finally, the analyses were conducted on 36 neonates in the protein supplement group and 36 in the control group (Figure 1).

Table 1 demonstrates the baseline characteristics of the neonates in both groups at birth and at the beginning of the intervention. As demonstrated, there were no significant differences among the baseline variables in the two groups (P>0.05). The participants’ mean GA was 30.34 weeks with a range of 26–33 weeks. The mean birth weight was 1220 grams with a range of 800–1400 grams. 16.7% of the participants in the control group and 19.4% in the protein supplement group were born small for gestational age (SGA). 52.8% of the participants were male, and 47.2% were female. The mean age of the neonates at the time of full enteral feeding either with FBM or PF was 14 days with a range of 5–23 days.

**Table 1.** Comparison of baseline characteristics of the neonates in the intervention and control groups before the clinical trial

**Variable**	**Groups**		P-value*

	**Protein supplement** **n=36**	**Control** **n=36**	
**Gender, n (%)**			0.345
Male	17 (47.2)	21 (58.3)	
Female	19 (52.8)	15 (41.7)	
**Birth weight (gr), mean ± SD**	1210.3 ± 189.53	1229.7 ± 169.6	0.660
**Birth weight percentile, n (%)**			0.798
≤ 3	3 (8.3)	1 (2.8)	
3 – 10	4 (11.1)	5 (13.9)	
> 10	29 (80.6)	30 (83.3)	
**Gestational age (weeks), mean ± SD**	30.50 ± 1.83	30.58 ± 1.70	0.850
**Gestational age groups (weeks), n (%)**			0.735
< 29	12 (33.3)	9 (25.0)	
29 – 31	11 (30.6)	12 (33.3)	
31 – 33	13 (36.1)	15 (41.7)	
**Birth head circumference (cm), mean ± SD**	26.6 ± 1.7	27.0 ± 1.5	0.600
**Birth length(cm), mean ± SD**	37.5 ± 17.63	37.3 ± 15.75	0.665
**Age (days), mean ± SD**	13.7 ± 9.0	11.6 ± 7.1	0.291
**Type of milk, n (%)**			1.000
Formula	19 (52.8)	19 (52.8)	
FBM	17 (47.2)	17 (47.2)	
**Type of formula, n (%)**			1.000
Pre BeBeLac	8 (47.1)	7 (38.9)	
Pre Aptamil	4 (23.5)	5 (27.8)	
Pre NAN	4 (23.5)	5 (27.8)	
BeBeLac AR	1 (5.9)	1 (5.6)	

SD: Standard deviation, FBM: Fortified breast milk, AR: Anti regurgitation

* Chi-square/Fishers’ exact test or independent sample t-test

Table 2 demonstrates the amount of protein intake, duration of trial, and the anthropometric parameters of the participants in the two groups. As demonstrated, the mean velocity of weight gain, weight on the discharge day, head circumference, and length growth velocity in the protein supplement group were significantly higher than the control group. Furthermore, based on the participant's weight percentile at the time of discharge, the number of patients under the third percentiles was significantly more frequent in the control group, while the patients above the 10^th^ percentile were significantly more in the intervention group. The incidence of postnatal growth failure was 75% and 61.1% in the control and protein supplement groups, respectively.

**Table 2.** The comparison of protein intake, duration of trial, and anthropometric parameters (outcomes) in the intervention and control groups

**Variable**	**Groups**		P-value*

	**Protein supplement** **n=36**	**Control** **n=36**	
**Protein Intake(gr/kg/day), mean ± SD**	4.55 ± 0.18	3.66 ± 0.22	<0.001
**Duration of trial (days), mean ± SD**	10.39 ± 4.42	12.50 ± 5.71	0.097
**Weight (gram), mean ± SD**			
Before intervention (gr)	1276.52 ± 127.23	1282.06 ± 118.30	0.854
After intervention (gr) (Discharge time)	1518.79 ± 59.78	1455.88 ± 50.38	**<0.001**
Gain (gr/kg/day)	19.12 ± 7.22	11.14 ± 5.29	**<0.001**
**Weight Percentile, n (%)**			**0.040**
**After intervention**			
≤ 3	6 (16.7)	16 (44.4)	
3 – 10	16 (44.4)	11 (30.6)	
> 10	14 (38.9)	9 (25.0)	
**Increased head circumference rate (cm/week), mean ± SD**	0.872 ± 0.262	0.525 ± 0.210	**<0.001**
**Increased length rate (cm/week), mean ± SD**	1.00 ± 0.35	0.62 ± 0.20	**<0.001**

SD: Standard deviation

* Chi-square/Fishers’ exact test or independent sample t-test

Bold values indicate significant p-value

Regarding the short time complications of protein supplement, blood gas analysis, BUN, and albumin levels were measured weekly, and the infants were observed for signs and symptoms of necrotizing enterocolitis (NEC). Table 3 demonstrates the laboratory data of the participants during the trial. The baseline levels of BUN, albumin, and VBG parameters were not significantly different among the two groups. BUN levels were not significantly different between the two groups and during repeated measurements. The serum albumin levels were significantly higher in the protein group and changed significantly throughout the study. The PH levels were significantly lower in the protein group, but the changes were not statistically significant throughout the study. None of the participants developed NEC during the hospital course.

**Table 3.** The comparison of laboratory data of neonates in the protein supplement and control groups during trial

**Test**	**Week**	**Groups**		P-value*

		**Protein Supplement** **(n=36)**	**Control group** **(n=36)**	
**BUN (mg/dl), mean ± SD**	Baseline	6.06 ± 1.39	6.71 ± 2.80	0.216
	1	5.56 ± 1.24	6.39 ± 2.39	0.069
	2	5.97 ± 1.21	5.84 ± 1.97	0.739
**Albumin (g/dL), mean ± SD**	Baseline	3.18 ± 0.33	3.13 ± 0.41	0.570
	1	3.41 ± 0.40	3.21 ± 0.37	**0.031**
	2	3.59 ± 0.37	3.24 ± 0.40	**0.000**
**PH, mean ± SD**	Baseline	7.34 ± 0.05	7.33 ± 0.06	0.044
	1	7.33 ± 0.04	7.36 ± 0.05	**0.006**
	2	7.33 ± 0.06	7.36 ± 0.04	**0.015**
**PCO_2_ (mmHg), mean ± SD**	Baseline	35.12 ± 6.54	35.97 ± 5.10	0.544
	1	39.20 ± 5.93	35.41 ± 5.95	**0.008**
	2	41.53 ± 6.68	34.63 ± 7.82	**0.000**
**HCO_3_ (mmol/L), mean ± SD**	Baseline	18.19 ± 4.33	18.39 ± 4.87	0.854
	1	20.47 ± 5.03	19.63 ± 4.72	**0.008**
	2	21.06 ± 5.25	19.58 ± 4.67	**0.004**

BUN: blood urea nitrogen; HCO_3_: Bicarbonate; PH: potential of hydrogen; SD: Standard deviation

* Independent sample t-test

Bold values indicate significant p value.

## Discussion

Preterm infants need to achieve their optimal growth rate, similar to intrauterine growth [12,18]. This aim is seldom achieved, and suboptimal postnatal growth still remains a significant complication in the VLBW infants. Moreover, there is a significant association between postnatal growth failure and adverse neurodevelopmental outcomes [5,6]. The present RCT trial evaluated the effect of extra protein supplement on the postnatal growth, and we compared the conventional protein intake (3.5 to less than 4 g/kg/day) with high protein intake (4–4.8 g/kg/day). The results showed that only 16.7% of the neonates in the control group and 19.4% of them in the protein supplement group were SGA, but the incidence of postnatal growth failure was increased during NICU admission (75% and 61.1% in the control and protein supplement groups, respectively). The incidence of PNGF in our control group was approximately similar to those reported by NICHD [3] and Gidi and colleagues [4], who reported 79% and 86% respectively. The incidence of PNGF declined about 14% by adding 0.89 g/kg protein per day. Likewise, the velocity of growth in all aspects considering weight, head circumference, and length were increased 7.98 g/kg/day, 0.347 cm/week, 0.38 cm/week, respectively. The intervention and control groups were entirely matched based on gender, GA, classified GA groups, birth time anthropometric parameters (weight, length and HC), classified birth weight percentile, type of feeding, and type of formula, as shown in Table 1. The means of weight, HC, and length at the time of discharge were significantly greater in the protein supplement group than the control group, as presented in Table 2. Our results are in the same line with the findings reported by Ergenekon and colleagues [16]. They assessed preterm infants with GA ≤32 weeks for the requirement of additional protein based on serum BUN and prealbumin levels after the standard early total parenteral nutrition and reaching full enteral feeding with 150–160 ml/kg/day of FBM or PF. They added additional enteral protein with BUN <5 mg/dl and/or prealbumin ≤8 mg/dl. There were 32 newborns in the non-supplemented group (Group 1) and 33 newborns in the supplemented group (Group 2). All newborns in Group 2 were on FBM. In this study, the mean weight gain velocity in Group 2 was significantly more than Group 1 (17 g/kg/day versus 11.5 g/kg/day). Linear growth in Group 2 was 0.9 cm/week, which was higher than group1 (0.7 cm/week), but this difference was not statistically significant. The rate of HC growth was 0.75cm/week in Group 2 and 0.6 cm/week in Group 1, which was significantly higher than Group 1 (p-value=0.007). They also checked Bayley Scales of Infant Development (BSID) at 18 months of corrected GA, which showed to be higher in Group 2. We utilized standard and targeted fortification, but they used adjustable fortification for titration of protein supplement. Similarly, protein supplementation improves the postnatal weight gain, linear growth, and HC growth in both studies. The velocity of growth in all aspects, however, was more in our infants (7.98 versus 5.5 gr/kg/day weight gain, 0.347 versus 0.15 cm/week HC growth, and 0.38 versus 0.2 cm/week linear growth). The main purpose of optimal nutrition in preterm neonates is to enable the best possible brain growth to obtain a favorable neurodevelopmental outcome in this vulnerable population. Saeidi et al. [19] followed 100 preterm and VLBW infants who were discharged from NICU; they did not evaluate the feeding pattern and the growth indices, but they reported that preterm infants had more disability and developmental delay. Our patients did not have long-term neurodevelopmental follow up, but according to Ehrenkranz's report [5] who showed infants with an average weight gain of 12 g/kg per day had significantly more neurodevelopmental impairment than those gained 21 g/kg per day, our neonates in the control group who had 11.14 g/kg/day weight gain were more vulnerable to neurodevelopmental delay than the protein supplement group with a weight gain of 19.12 g/kg/day. Our patients also had significantly more linear growth in the protein supplement group which, considering Ramel's report [6], get better neurodevelopmental outcomes. In line with our data, Arslanoglu and colleagues [20] also showed a significant improvement in weight gain with higher protein supply adjusted according to the serum level of BUN. They, however, evaluated lower levels of protein intake than those of our study (2.8 vs 3.4 g/kg/d protein intake). Atchley et al. [21] also showed that increased protein intake led to increased weight gain in premature infants. They assessed body composition by using air displacement plethysmography, showing that the fat mass increased with augmented protein intake. Contrary to our results, Maas et al. [17] and Miller et al. [22] found no effect of increased enteral protein intake on weight gain. Maas and colleague hypothesized that their findings indicated a potential ceiling effect for enteral protein supply and that intake exceeding 3.5 to 4.0 g/kg/d might not further improve the weight gain in preterm infants. They supported their hypothesis by the finding of increased concentration of BUN in the higher-protein group, indicating that additional protein was metabolized to urea instead of being used for body protein synthesis [17]. In contrast to the data reported by Maas et al., our results showed that the difference in the BUN levels was not significant between the protein supplement and control groups. Also, based on repeated measures analysis, the changes in BUN during the study were not statistically significant. An increase in BUN levels indicates that the anabolic potential of proteins is not used completely. Numerous factors such as acute sepsis, poor-quality dietary proteins or amino acids, and inadequate intake of calories to sustain lean tissue accretion may contribute to this [23]. These factors may cause the lack of effect of additional protein on the weight gain in the Maas and Miller's studies. Furthermore, we checked the serum albumin levels which were significantly higher in the protein supplement group and increased significantly in this group throughout the study (based on repeated measures analysis). In the Cochrane review published at 2020 [24] which evaluated high versus low protein intake in the formula-fed LBW infants, no study addressed serum albumin as a secondary outcome; however, in studies which compared very high versus low protein intake, Cooke et al. [25] reported serum albumin levels and found no significant differences between the two groups. Van der Aker et al. reported that albumin synthesis was stimulated by parenterally administrated amino acid in preterm infants [26]. The increase in serum albumin in our neonates can indicate the anabolic effect of extra protein and increased weight gain velocity is due to protein and fat accretion. One putative risk for high protein intake is increased concentrations of hydrogen ions as a result of the immaturity of amino acid metabolic pathways in preterm infants [27], and metabolic acidosis may develop. Hence, we evaluated the acid-base status of the participants. The PH was significantly lower, while PCO2 and HCO3 were higher in the protein group than the controls (after intervention). Based on repeated measures analysis, however, the changes in the PH level and HCO3 levels were not statistically significant throughout the study in both groups. Thus, no significant metabolic acidosis developed, like the result of Cooke et al.'s study [25].

Our study had some limitations such as a lack of long-term follow-up of our patients and the small sample size. However, based on group matchings and insignificant differences between the baseline features of our groups, we believe that our study could provide insight into further multicentral RCTs and review of articles to obtain the most satisfactory treatment and supplement regimen for neonates.

## Conclusion

Protein supplement significantly improved the growth of all aspects of the anthropometric parameters including weight, length, and head circumference. No short-term adverse effect was detected during the study. This study could provide insight into further multicenter RCTs to define the optimal levels of protein intake for VLBW neonates and add protein supplement to the routine feeding protocols. Furthermore, long-term studies are needed to evaluate long-term benefits, especially on neurodevelopment and probable complications of this intervention.

## Abbreviations

GA: Gestational Age
BUN: Blood Urea Nitrogen
VLBW: Very Low Birth Weight
NEC: Necrotizing Enterocolities
SGA: Small for Gestational Age
HC: Head Circumference
FBM: Fortified Breast Milk
PF: Premature Formula
PNGF: Post Natal Growth Failure
NICDH: National Institute of Child Health and Human Development
PDA: Patent Ductus Arteriosus
MOM: Mothers’ Own Milk
AAP: American Academy of Pediatrics
ESPGHAN: European Society for Pediatric Gastroenterology Hepatology and Nutrition
